# Die Weiterbildungssituation in der HNO-Heilkunde in Deutschland

**DOI:** 10.1007/s00106-020-00838-9

**Published:** 2020-03-04

**Authors:** J. Linke, T. Eichhorn, M. Kemper, T. Zahnert, M. Neudert

**Affiliations:** 1 Klinik für Hals‑, Nasen- und Ohrenheilkunde, Universitätsklinikum Carl Gustav Carus Dresden, Fetscher Str. 74, 01307 Dresden, Deutschland; 2 Emeritus HNO-Klinik Carl-Thiem-Klinikum, Cottbus, Deutschland

**Keywords:** HNO-Facharztweiterbildung, Hals-Nasen-Ohren-Heilkunde, Evaluation der Weiterbildung, Assistenzarztausbildung, Weiterbildungsstruktur, ENT residency training, Otorhinolaryngology, Evaluation of residency training, ENT resident, Residency training structure

## Abstract

**Hintergrund:**

Als Ansatzpunkt zur Entwicklung konkreter Optimierungsvorschläge für das HNO-ärztliche Weiterbildungssystem wurde zunächst die Ausbildungssituation an deutschen HNO-Kliniken analysiert, um existierende Schwachstellen der Weiterbildung identifizieren zu können.

**Methodik:**

HNO-Weiterbildungsassistenten an deutschen HNO-Kliniken wurden eingeladen, an einer Online-Befragung teilzunehmen. Der Fragebogen bestand dabei aus 78 Einzelfragen.

**Ergebnisse:**

An der Umfrage nahmen 223 Weiterbildungsassistenten der HNO-Heilkunde teil. Defizite der Ausbildung wie ein fehlendes regelmäßiges Feedback zwischen Weiterbildungsberechtigten und Weiterbildungsassistenten/-innen, eine nur mittelmäßig bewertete Vermittlung von Fachkompetenz sowie eine in den Freitextkommentaren genannte „Willkür“ der Weiterbildungsbeauftragten hinsichtlich der Gestaltung des Weiterbildungsverlaufs sowie Zeit- und Personalmangel treten in den Fokus unserer Auswertung. Einige der verbindlich aufgeführten Empfehlungen der Weiterbildungsordnung wie die Festlegung von konkreten Weiterbildungszielen im Rahmen eines regelmäßigen Gesprächs oder der Einsatz eines Log-Buchs werden bislang nur vereinzelt umgesetzt. Forderungen zum Ausbau von externen Fortbildungsmöglichkeiten (Hospitation/Rotation) und einer externen objektiven Überprüfung der klinikinternen Durchführung der Weiterbildung im Sinne einer Qualitätssicherung beispielsweise durch Fachvertreter der Deutschen Gesellschaft für HNO-Heilkunde, Kopf- und Halschirurgie (DGHNOKHC) werden diskutiert.

**Schlussfolgerung:**

Die Umsetzung einer strukturierten und standardisierten Weiterbildung in Deutschland, welche im Sinne einer freiwilligen Eigeninitiative auch durch die DGHNOKHC in ihrer Umsetzung überprüft wird, kann eine Basis für eine effektivere verbindliche Weiterbildung schaffen.

**Zusatzmaterial online:**

Die Online-Version dieses Beitrags (10.1007/s00106-020-00838-9) enthält eine Tabelle, die Ergebnisse einer Fragenauswahl gegliedert nach Themenkomplexen darstellt. Beitrag und Zusatzmaterial stehen Ihnen auf www.springermedizin.de zur Verfügung. Bitte geben Sie dort den Beitragstitel in die Suche ein, das Zusatzmaterial finden Sie beim Beitrag unter „Ergänzende Inhalte“.

## Abstract (en)

**Background:**

As a starting point for developing concrete suggestions for optimizing specialty training in German ENT departments, the present study analysed the current status of residency training in order to identify weaknesses.

**Methods:**

Residents at German ENT departments were invited to participate in an online survey. The questionnaire comprised 78 individual questions.

**Results:**

The questionnaire was answered by 223 ENT residents. Educational deficits such as a lack of regular feedback from supervisors, only moderately rated mediation of specialist competencies, capriciousness of the trainers in charge regarding the organisation of training, and time and personnel shortages were identified. Some of the mandatory recommendations of the specialty training regulations, including determination of concrete training goals during regular meetings or the use of a logbook, have only been implemented in limited cases. Demands for development of more external training opportunities (job shadowing/rotation) and an external objective review of the residency training (e.g., by a representative of the German Society of Otorhinolaryngology, Head and Neck Surgery, DGHNOKHC) in the sense of quality assurance are discussed.

**Conclusion:**

Implementation of structured and standardized ENT residency training in Germany, for which DGHNOKHC reviewal is also possible, can generate a basis for more effective compulsory residency training.

Die Zeit der ärztlichen Weiterbildung ist eine wichtige und entscheidende Phase im beruflichen Werdegang junger Ärzte. In ihr durchlaufen sie alle Stationen, die aus einem frisch approbierten Hochschulabsolventen einen fähigen und in allen Bereichen kompetenten Facharzt machen können. Dabei stellt insbesondere die erste Schnittstelle zwischen der Beendigung des Studiums und dem Einstieg in den Beruf eine erhebliche Zäsur in der Ausbildungsbiographie der Ärzte dar. Sie verlieren mit ihrem Studentenstatus auch zuvor die meist gut organisierte, wohl geplante und aufeinander abgestimmte curriculare Struktur der Wissens- und Fertigkeitenvermittlung an den Kliniken der Medizinischen Fakultäten.

Im Gegensatz zu anderen Ländern (wie beispielsweise USA und UK), in denen die Facharztweiterbildung in Form staatlich organisierter und kontrollierter Programme stattfindet, ist die Durchführung der ärztlichen Weiterbildung in Deutschland von den staatlichen Gesundheitsbehörden (Gesundheitsministerien der Bundesländer) an die Landesärztekammern delegiert. Diese haben die regulatorischen Vorgaben der Bundesärztekammer (Weiterbildungsordnung) auf Länderebene umzusetzen. Damit handelt es sich um ein Organisationsmodell „von Ärzten – für Ärzte“, welches die Weiterbildungshoheit auf persönlich ermächtigte Weiterbildungsberechtigte (WBB) verteilt. Dadurch liegt die Qualität der Facharztweiterbildung formal weitgehend in der Hand des Weiterbildungsbeauftragten, wie auch in vielen anderen europäischen Ländern (Österreich, Spanien, Italien, Frankreich, Belgien).

In einer 2009 erstmals durch die Bundesärztekammer (BÄK) zusammen mit den Landesärztekammern durchgeführten, bisher umfangreichsten Befragung zur bundesweiten Weiterbildungssituation gaben die Weiterbildungsassistenten (WBA) ihren Weiterbildungsstätten (WBS) mit einer Globalbeurteilung von 2,54 („Ich würde die WBS weiterempfehlen“, „Ich bin zufrieden mit der Arbeitssituation“), insgesamt gute Noten (Bewertung identisch wie im Schulnotensystem) [1]. Dabei lag die Beurteilung der Weiterbildungssituation in der Untergruppe „FA Hals-Nasen-Ohren-Heilkunde“ (*n* = 308) nahezu im bundesdeutschen Durchschnitt. Allerdings zeigte die Umfrage auch, dass lediglich der Hälfte aller WBA ein strukturierter Weiterbildungsplan zur Verfügung gestellt wurde, und 40 % der befragten WBA gaben an, dass mit ihnen keine Lern- und Weiterbildungsziele vereinbart werden [2]. Diese und weitere Punkte, die zu einer Unzufriedenheit mit der Weiterbildungssituation in Deutschland führen, werden u. a. mit als Gründe für die zur Zeit zunehmend beobachtete Abwanderung des ärztlichen Nachwuchses in das Ausland verantwortlich gemacht [3–5].

Wenngleich die HNO-Heilkunde in Deutschland laut Studien weniger stark vom drohenden Fachkräftemangel betroffen zu sein scheint als andere Fachrichtungen [6], darf dieses Ergebnis nicht zu einer Unterschätzung der Problematik führen. Vielmehr scheint eine gewisse Dringlichkeit hin zu einer grundsätzlichen freiwilligen Neustrukturierung der Weiterbildungsbedingungen geboten, um ein zukünftiges Übergreifen des Fachkräftemangels auch auf das HNO-Fachgebiet zu verhindern.

Um einen möglichst dezidierten fachgruppenspezifischen Status quo der Weiterbildungssituation in der HNO-Heilkunde zu erheben, erfolgte auf Initiative der Arbeitsgruppe „Vorbereitung zum HNO-Facharzt“ der Deutschen Gesellschaft für HNO-Heilkunde, Kopf- und Halschirurgie (DGHNOKHC) eine Umfrage unter den WBA. Die Ergebnisse sollten dabei zur Identifikation von Problembereichen dienen, die wiederum einerseits die Grundlage für die Entwicklung von Verbesserungsvorschlägen darstellen und andererseits als Ausgangspunkt für weitere, später nachfolgende Befragungen ähnlicher Art Verwendung finden können.

## Methodik

In Anlehnung an die bereits 2009 fächerübergreifend erfolgte Umfrage der BÄK zur „Evaluation der Weiterbildung in Deutschland“ wurde von uns ein neuer Fragebogen konzipiert. Dieser Online-Fragebogen wurde anonymisiert den an deutschen HNO-Kliniken angestellten WBA in der Zeit von 12/2010 bis 3/2011 zur Verfügung gestellt (EvaSys Electric Paper, Lüneburg). Zugang zu diesem Online-Fragebogen erhielten die WBA über einen Teilnahmecode, der den deutschen Klinikdirektoren und Chefärzten über den Postverteiler der DGHNOKHC in Bonn sowie über die in der Assistentenvertretung der DGHNOKHC organisierten Assistenzärzte zugesandt wurde.

Dabei wurden 40 HNO-Abteilungen an Universitätskliniken und 113 HNO-Abteilungen in Deutschland angeschrieben. Neben einem Anschreiben an die einzelnen Klinikdirektoren und einem Brief für die Assistentenvertreter lagen dem Schreiben bis zu 20 Transaktionsnummern (TAN) für den Onlinezugang zum Fragebogen bei. Die Klinikdirektoren wurden gebeten, diese TAN an die WBA weiterzureichen. Zur Erhöhung der Rücklaufquote erfolgte 2 Monate nach Beginn der Datenerhebung eine erneute schriftliche Kontaktaufnahme mit den Klinikdirektoren, in denen an diese die Bitte gerichtet wurde, ihre WBA nochmals auf die Teilnahme an der Befragung hinzuweisen. Inhaltlich war der Fragebogen stark an den der BÄK angelehnt [7]. Es wurden die Themenbereiche: beruflicher Hintergrund, Lernkultur, Eigenaktivität, Ausbildungsstruktur, Vermittlung von Fachkompetenz, Führungs- und Betriebskultur sowie die Arbeitssituation abgefragt. Insgesamt wurden 78 Aussagen (Items) als Beurteilungskriterien formuliert und von den Teilnehmern auf einer 6‑stufigen Likert-Skala, analog dem deutschen Schulnotensystem gewichtet (1 „stimme zu/sehr gut“ bis zu 6 „stimme nicht zu/sehr schlecht“), abgefragt. Zudem bestand die Möglichkeit zu Äußerungen in Form von Freitextkommentaren (FTK). Die Auswertung erfolgte mit der systemeigenen Software (EvaSys Electric Paper, Lüneburg) unter Berechnung der Mittelwerte und Standardabweichungen. Die FTK wurden inhaltlich analysiert und in Antwortkategorien eingeteilt.

Die Ergebnisauswertung erlaubte keine Zuordnung der versendeten TAN zu dem jeweils beantworteten Fragebogen. Die Bildung von Untergruppen in der Auswertung war nur anhand der in dem Fragebogen erfassten Kenndaten möglich, wodurch die Anonymität der Umfrage gewährleistet war.

## Ergebnisse

### Strukturelle Ergebnisse

An der Befragung nahmen 223 WBA teil. Eine genaue Gesamtzahl der zu dem Erhebungszeitraum in deutschen Kliniken tätigen HNO-WBA ist aufgrund der Tatsache, dass das Statistische Bundesamt seit einer Novellierung der Krankenhausstatistik-Verordnung die Anzahl von WBA nicht mehr bei den Krankenhäusern erhebt, nicht verfügbar [2, 4]. Nach Angaben der Gesundheitsberichterstattung des Bundes beenden jährlich 191 (106 M./89 F., 2011) bzw. 218 (112 M./106 F., 2012) HNO-Ärzte ihre Facharztausbildung [8]. Somit kann eine Anzahl von 800–900 WBA angenommen werden [9]. Damit kann man von einer Rücklaufquote zwischen 25 bis 28 % ausgehen.

**Figure 1:**
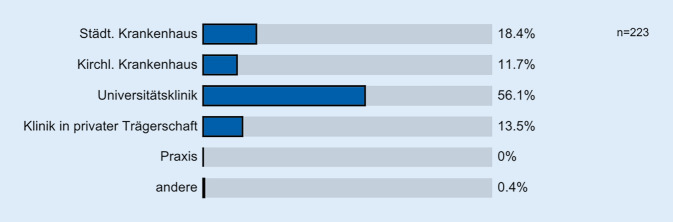

Das Geschlechtsverhältnis war mit 49,8 % weiblichen und 50,2 % männlichen Befragten ausgeglichen. Es ergab sich ein Durchschnittsalter der WBA von 31,32 Jahren. Mit einem Anteil von 56,1 % (*n* = 125) sind die derzeit an Universitätskliniken beschäftigten WBA in dieser Befragung überrepräsentiert (Abb. 1: 13,5 % Kliniken in privater Trägerschaft, 18,4 % kommunale Krankenhäuser, 11,7 % kirchliche Krankenhäuser, 0,4 % andere). Vertreter aus den einzelnen Weiterbildungsjahrgängen waren mit jeweils rund einem Fünftel gleich häufig repräsentiert.

### Inhaltliche Ergebnisse

Im Folgenden sollen die wichtigsten Kernaussagen der einzelnen Fragekomplexe herausgestellt werden. Dazu sind in Tab. 1 (elektronisches Zusatzmaterial) die Ergebnisse einer Fragenauswahl gegliedert nach Themenkomplexen dargestellt. Die vollständige Auswertung des Fragebogens ist unter http://tinyurl.com/Fragenauswahl oder über den QR-Code abrufbar.

#### Beruflicher Hintergrund

Obwohl eine Verlängerung des zeitlich befristeten Arbeitsvertrags bei 58,9 % (*n* = 123) der WBA ungewiss ist, gaben 73,3 % (*n* = 162) aller befragten Assistenzärzte an, eine berufliche Perspektive („Ja“ und „Ja, jedoch mit Einschränkungen“) an ihrer jetzigen Weiterbildungsstätte zu sehen.

Insgesamt würden 80,2 % (*n* = 174) der Befragten ihre Weiterbildungsstätte auch weiterempfehlen. Im Einzelnen entspricht das 77,9 % der Befragten an Universitätskliniken, 83,3 % an Kliniken in kirchlicher und privater Trägerschaft und 82,5 % der Befragten an städtischen Einrichtungen.

#### Lernkultur

Defizite wurden v. a. hinsichtlich der kommunikativen Aspekte im Rahmen der Weiterbildung deutlich. Die Vermittlung eines regelmäßigen Feedbacks seitens der Weiterbildungsberechtigten wurde mit 4,0 ± 1,5 bewertet, und auf die Frage, ob sich der WBB ausreichend Zeit nimmt, um Fragen zu beantworten/Zusammenhänge zu erklären, ergab sich eine Benotung von 3,3 ± 1,6. Die Einstufung, welcher Wert einer klinikinternen Lehre beigemessen wird („Ist es an Ihrer Weiterbildungsstätte ein wichtiges Ziel, eine gute Weiterbildung zu bieten?“), wurde mit 3,3 ± 1,6 mittelmäßig bewertet.

#### Eigenaktivität

Die Eigeninitiative der befragten WBA hinsichtlich des Erwerbs zusätzlicher Qualifikationen und das Ausmaß an Akquise von Wissen außerhalb des Klinikbereichs sowie die aktive Teilnahme an der Weiterbildung wird laut eigenen Aussagen der WBA sehr hoch eingeschätzt: 87,3 % (*n* = 192) der WBA besuchen mindestens halbjährlich zusätzliche Fort- und Weiterbildungsveranstaltungen (25,5 % wöchentlich), und 85,4 % (*n* = 187) beteiligen sich regelmäßig mit einem eigenen Beitrag an den klinikinternen Fort- und Weiterbildungsveranstaltungen. 88,3 % (*n* = 106) der Befragten an Universitätskliniken und 35,4 % (*n* = 34) von denen an nichtuniversitären Häusern geben als Ziel an, wissenschaftliche Arbeiten verfassen und publizieren zu wollen, wobei hierfür, unabhängig von der Trägerschaft der Klinik, nur 22 % zeitliche Freiräume in der Regelarbeitszeit zur Verfügung gestellt bekommen.

#### Ausbildungsstruktur

**Figure 2:**
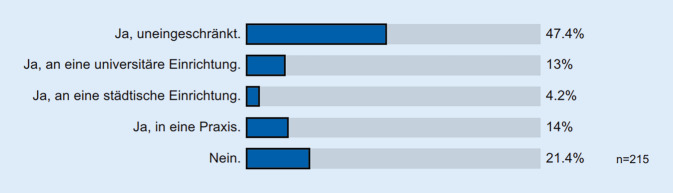

**Figure 3:**
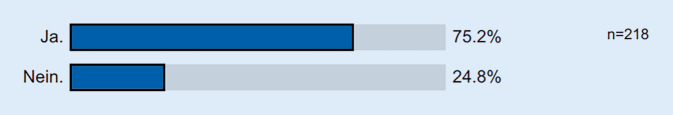

Die Zufriedenheit mit der aktuellen Ausbildungssituation wurde insgesamt mit 3,0 ± 1,5 bewertet, wobei sie in den Unikliniken mit 3,2 ± 1,4 am schlechtesten eingeschätzt wurde (private Krankenhäuser, KH: 2,5 ± 1,3; kirchl. KH: 2,6 ± 1,2; komm. KH: 3,1 ± 1,6). Das in der Weiterbildungsordnung geforderte jährliche Gespräch mit dem WBB findet nur bei 44,8 % (*n* = 99) der Befragten regelmäßig statt [10]. Bei 50,7 % der Befragten erfolgt die Ausbildung ohne Einsatz eines Logbuchs. Nur 21,2 % der WBA wurde ein strukturierter Weiterbildungsplan zur Kenntnis vorgelegt. Eine Weiterbildungsstättenrotation wird von 78,6 % der WBA befürwortet (Abb. 2) und eine Hospitation an anderen Weiterbildungsstätten von 89,5 % gewünscht. Es sprachen sich 64,1 % (*n* = 143) der Befragten für die Möglichkeit einer solchen Weiterbildungsstättenrotation aus, diese sollte jedoch fakultativ bleiben und nicht als verbindlicher Bestandteil in die Weiterbildungsordnung aufgenommen werden. Eine externe Überprüfung der klinikinternen Weiterbildung durch eine neutrale, übergeordnete Institution, namentlich der DGHNOKHC, wird von 75 % der Befragten gewünscht (Abb. 3).

Die in der Weiterbildungsordnung festgelegten Ziele werden in der Regelweiterbildungszeit bei 34,3 % (*n* = 73) der Befragten WBA vollständig und bei 49,8 % (*n* = 106) nur teilweise bzw. in 16 % (*n* = 34) der Fälle nur ungenügend erreicht.

#### Vermittlung von Fachkompetenz

Die praktische Weiterbildung wird bei 86,5 % (*n* = 193) der WBA hauptsächlich von einem Oberarzt, bei 52,5 % durch einen Facharzt, bei 37,8 % durch einen älteren Assistenzarzt und nur bei 22,0 % durch den Chefarzt selbst betreut (Mehrfachnennungen möglich). Immerhin gaben 11,7 % (*n* = 26) der Befragten auch an, ihre praktische Weiterbildung würde gar nicht betreut werden. Diese letztgenannte Antwort erfolgte von Assistenzärzten an Kliniken in privater Trägerschaft in keinem einzigen Fall. Hier wird die praktische Ausbildung nahezu immer von einem Oberarzt (96,7 %) fachlich begleitet.

Zusätzlich wurde die Vermittlung von Fertigkeiten wie die Anleitung zum wissenschaftlichen Arbeiten, das Erlernen von Untersuchungstechniken und die Befundauswertung usw. erfragt. Der daraus gebildete Score ergab insgesamt die Note 3,0 ± 1,4. Besonders schlecht wurde dabei die Anleitung zum wissenschaftlichen Arbeiten mit 4,2 ± 1,5 bewertet. Dazu kontrastiert mit einer Bewertung von 2,2 ± 1,2 die Übertragung von Verantwortung/selbstständiges Arbeiten an den WBA.

#### Führungs- und Betriebskultur

Das allgemeine Arbeitsklima wurde insgesamt mit 2,7 ± 1,4 eingestuft, wobei keine ausgeprägten Unterschiede hinsichtlich der Trägerschaft der Kliniken beobachtet werden konnten. 46,4 % der Befragten stimmten „voll“ (1) bis „überwiegend“ (2,3) der Aussage zu, dass „Kritik an Ihrer Arbeitsweise angemessen formuliert werde“. Insgesamt wurde dieser Aussage mit 3,0 ± 1,5 zugestimmt (1 – stimme zu bis 6 – stimme nicht zu). 50,2 % bestätigten, dass „in der Abteilung auf eine angemessene Kommunikation Wert gelegt werde“.

#### Arbeitssituation

Die Arbeitsbelastung („Ich bin insgesamt zufrieden mit der jetzigen Arbeitsbelastung.“) wurde mit 3,4 ± 1,6 bewertet, wobei die Noten zwischen Assistenten an den Kliniken in privater Trägerschaft einerseits (2,8 ± 1,6) und denen an kommunalen Häusern (3,6 ± 1,7) andererseits deutlich divergierten. Angegeben wurde, dass in der vertraglich geregelten Arbeitszeit weder die Weiterbildung noch die tatsächlich abgeforderte klinische Tätigkeit zur eigenen Zufriedenheit erfüllt werden können (3,8 ± 1,7 und 3,7 ± 1,6). Die personelle Ausstattung wurde mit 3,1 ± 1,4 bewertet. Der Hinweis auf die Problematik, dass die im Laufe der Zeit kontinuierlich zugenommenen administrativen Aufgaben eine erhebliche Belastung der täglichen Arbeit bedingen, erfuhr mit 2,2 ± 1,4 eine deutliche Zustimmung.

**Figure 4:**
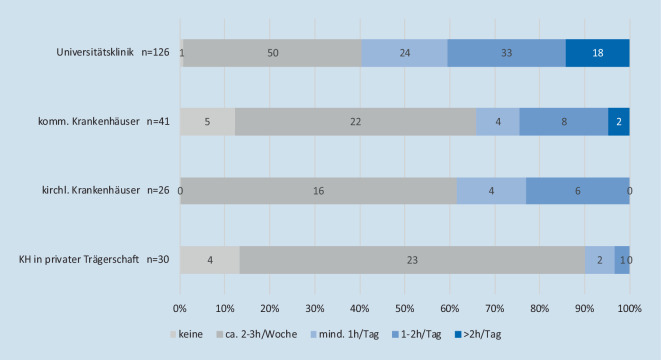

Die Verteilung der durchschnittlich geleisteten Mehrarbeit/Überstunden wird in Abb. 4 dargestellt, wobei die Mehrstunden an Arbeitseinsatz nur bei 42,1 % der Befragten vollständig bzw. bei 36,6 % teilweise dokumentiert werden. Ein Ausgleich der geleisteten Überstunden erfolgt zu 65,9 % in Form von Freizeitausgleich, in 32,7 % der Fälle wird dieser nicht gewährt.

#### Freitextkommentare

In 83 Freitextkommentaren (FTK) wurde überwiegend die fehlende objektive Strukturierung der Weiterbildung (45 FTK) und die daraus für den Einzelnen resultierende „Beliebigkeit“ in ihrem Weiterbildungsprozess bemängelt (12 FTK). Zudem wurde von den Befragten darauf hingewiesen, dass vermehrt und regelmäßig strukturierte und bindende Zielvereinbarungen bezüglich des Weiterbildungsablaufs zwischen ihnen und den WBB erfolgen mögen (45 FTK). Neben dem Wunsch nach einer strukturierteren innerklinischen Fortbildung mit festgelegten Ausbildungsstandards wurde v. a. auch das Bedürfnis nach einem verstärkten Feedback (11 FTK) zwischen WBA und WBB sowie eine intensivierte direkte, persönliche Ausbildungsanleitung durch einen Mentor/Oberarzt geäußert (16 FTK). Ursächlich und als wünschenswert verbesserungsbedürftig wurde ferner der durch die vermehrte Bürokratisierung und die geringe Personaldichte entstehende Zeitmangel zur Verrichtung der klinischen Arbeiten erachtet (35 FTK).

## Diskussion

Die Ergebnisse der vorliegenden Studie skizzieren die Einstellung und Stimmung der WBA hinsichtlich ihrer aktuellen Arbeits- und Weiterbildungssituation im Jahr 2010/2011. Die Zeitspanne zwischen Datenerhebung und Publikation der Ergebnisse reduziert natürlich die Aussagekraft im Hinblick auf ihre Aktualität. Trotzdem stellen die Ergebnisse nach unserer Auffassung eine wertvolle und weiterhin nutzbare Datengrundlage dar, da es sich

1. um die erste HNO-spezifische Datenerhebung auf nationaler Ebene handelt und
2. diese als Ausgangspunkt für nachfolgende Umfragen zur Weiterbildungssituation betrachtet werden kann.

In der Zwischenzeit wurde seitens der BÄK (BÄK) eine neue Musterweiterbildungsordnung (MWBO) erarbeitet und auf dem Bundesärztetag 2018 in Erfurt angenommen [11]. Diese hat sich eines Teils der auch von uns aufgelisteten Mängel in der bisherigen Weiterbildung angenommen und hält für deren Beseitigung Lösungsvorschläge parat (z. B. elektronisch geführtes Logbook, Möglichkeit von Rotationen, Weiterbildungsinhalte in Kompetenzblöcke untergliedert, Dokumentation der jährlichen „Feedbackgespräche“), scheint jedoch bei Weitem nicht für alle Unzulänglichkeiten passende Verbesserungen anzubieten. Die Umsetzung dieser neuen MWBO in den einzelnen Landesärztekammern wird in naher Zukunft erfolgen. Erst bei deren praktischer Umsetzung wird zu sehen sein, inwieweit sie in der Lage ist, zu positiven Fortschritten in dem Weiterbildungssystem beizutragen.

Es darf zunächst als positives Zeichen der Studie gewertet werden, dass 73,3 % der befragten WBA an ihrer aktuellen Weiterbildungsstätte, mit kleineren oder ohne Einschränkungen eine berufliche Perspektive sehen und ganze 80,2 % ihre WBS auch weiterempfehlen würden. Dieses zunächst als gut oder befriedigend anzusehende Ergebnis sollte jedoch einer differenzierteren Betrachtung unterworfen werden.

Die Zufriedenheit mit der Ausbildungsstruktur wurde immerhin nur mit der Note 3,0 bewertet, was Anlass zu weiteren Überlegungen gibt. Damit liegt die Bewertung aber immerhin deutlich über dem 2014 von Oker et al. erhobenen Wert von 5,3 (Skala von 0–10) [9]. Es scheint naheliegend, dass die Gründe hierfür u. a. in der teilweise als unzureichend empfundenen oder real sogar fehlenden Kommunikation zwischen dem Weiterbilder und Weiterzubildendem liegen. Mehr als 2 Dritteln der WBA liegt kein strukturierter Weiterbildungsplan vor, und ein die Weiterbildung begleitendes Logbuch wird auch nur in knapp der Hälfte der Fälle im Rahmen der Weiterbildung verwendet. Zudem geben nur 44,9 % der Befragten an, dass das in der Weiterbildungsordnung eigentlich festgelegte jährliche Gespräch mit ihrem WBB auch wirklich regelmäßig stattfindet [11, 18]. Vor diesem Hintergrund scheint die lediglich als „befriedigend“ eingeschätzte Beurteilung durchaus gerechtfertigt, werden doch anscheinend in der Mehrzahl der Fälle einige klare Vorgaben und Verpflichtungen der Weiterbildungsordnung nicht ausreichend ernst genommen oder aber anderweitig bereits existierende Instrumente zur Strukturierung der 5‑jährigen Weiterbildung nicht ausreichend genutzt [13–15]. Diese Daten werden durch den vorgetragenen Wunsch der WBA nach einem regelmäßigen und detaillierten Feedback (Note 4,0) bezogen auf ihre Leistungseinschätzung und der deutlich verstärkt gewünschten Vermittlung angemessener Kritik an ihrer Arbeitstätigkeit weiter unterstrichen. Auch wenn anhand der erhobenen Daten nicht zwischen der eher dürftigen Quantität auf die Qualität des gegebenen Feedbacks unterschieden werden kann, ergibt sich aus unserer Sicht für diese Form der gelebten Weiterbildungskultur doch ein stark verbesserungswürdiges Potenzial. Indirekt hingegen, so glauben wir, können aus den von uns erhobenen Daten doch sogar vorsichtig Rückschlüsse auch über die Qualität des Feedbacks gezogen werden, denn nur 46,4 % der Befragten stimmten „voll“ oder „überwiegend“ der Aussage zu, dass „Kritik an Ihrer Arbeitsweise angemessen formuliert werde“ (Durchschnittsnote 3,0), und nur 50,2 % bestätigten in analoger Weise, dass „in der Abteilung auf eine angemessene Kommunikation Wert gelegt werde“. Der Bedarf und die Wichtigkeit zur Optimierung der Fehlerkultur wurde auch in der durch die BÄK initiierten Befragung der WBA 2009 und 2011 zum Ausdruck gebracht [2, 3]. Dabei wurde die „Kultur zur Fehlervermeidung“ im Schulnotensystem 2009 mit 2,81 und 2011 mit 2,67 bewertet. Abgefragt wurde dabei der offene Umgang mit Fehlern, Vorhandensein eines CIRS (Critical Incident Reporting System) und ob eine Zwischenfallmeldung zur Prozessverbesserung führt.

HNO-WBA gaben an, dass die in der Weiterbildungsordnung festgelegten Ziele üblicherweise nur in 34,3 % der Fälle „vollständig“ und in 49,8 % „teilweise“, bzw. in 16,0 % in „ungenügendem Maße“ in der Regelweiterbildungszeit erreicht werden. Daher verwundert die sogar noch nur befriedigende Beurteilung (Note 3,3) der Frage, ob es für die WBA erkennbar sei, dass die Weiterbildung in ihrer Abteilung zu einem wichtigen Ziel erklärt sei, keineswegs. Ähnliche Ergebnisse wurden auch von Oker et al. im europäischen Vergleich (Spanien, Frankreich, Italien, Österreich und Belgien) gefunden [9, 12].

Demgegenüber scheint sich bei den WBA eine hohe Bereitschaft und zeitliche Investitionsfreudigkeit, zumindest aufgrund ihrer eigenen Angaben, für die eigene Weiterbildung abzuzeichnen. Neben der regen Teilnahme an Weiterbildungsveranstaltungen bereichern immerhin 85,4 % der Weiterzubildenden regelmäßig durch einen eigenen Beitrag die klinikinternen Fort- und Weiterbildungsmaßnahmen. Als Schlussfolgerung lässt sich daraus ableiten, dass demnach bereits ein nicht unerheblicher Anteil der Weiterbildungsveranstaltungen, der hauptsächlich für die WBA gedacht ist, durch eben diese Gruppe der WBA selbst abgedeckt und nicht durch die erfahrenen und zur Weiterbildung ermächtigten Fach‑, Ober- oder Chefärzte gestaltet wird. Dabei kann man den Wert einer intensiven Auseinandersetzung mit einer relevanten Thematik für den eigenen Wissenserwerb, wie er bei dem WBA während der Erarbeitung eines Themenkomplexes für eine innerklinische Präsentation zum Tragen kommt, gemäß dem bereits durch Seneca benannten Lehrsatz „Docendo discimus“ (Durch das Lehren lernen wir.) unbestritten als hoch einstufen. Dennoch sollte es nach Meinung der Autoren nicht zu einer Verlagerung der Weiterbildungsbemühungen in den Bereich des Selbststudiums der WBA kommen.

Der Wunsch der WBA nach einer umfassenden und fundierten Weiterbildung zeigt sich auch darin, dass 47,4 % der Befragten eine Weiterbildungsstättenrotation uneingeschränkt für sinnvoll halten und 58,9 % eine solche, zumindest verbal formuliert, auch in Anspruch nehmen würden. Ganze 89,0 % würden es befürworten, wenn „auf dem Markt“ mehr direkt auf die Facharztprüfung ausgelegte Seminare und Kurse angeboten würden, was sich auch an der regen Nachfrage, zahlreichen Beteiligung und regelmäßigen Überbelegungen der Kurse der deutschen Akademie für HNO-Heilkunde e. V. widerspiegelt. Zwischenzeitlich, so kann man konstatieren, sind auf diesem Sektor doch einige zusätzliche Angebote hinzugekommen, die sich speziell der Vorbereitung auf die Facharztprüfung beziehen. In diesen mehrtägigen Kompaktseminaren oder Rubrikspalten der gängigen Fachzeitschriften wird versucht, möglichst umfassend die prüfungsrelevanten Stoffinhalte zu vermitteln.

Im Gegensatz zu den in Deutschland an einzelne Personen vergebenen Weiterbildungsermächtigungen, die in der Regel an den Klinikdirektor oder Chefarzt einer Abteilung gebunden ist, wird es von den WBA so wahrgenommen, dass die im Alltag vermittelte praktische Weiterbildung in 86,5 % schwerpunktmäßig durch einen Oberarzt oder Facharzt erfolgt. Diese gängige Praxis darf dabei durchaus nicht von vornherein als Qualitätseinbuße interpretiert werden. Allein von dem administrativen und klinischen Aufgabenumfang, den ein Klinikleiter zu bewältigen hat, erscheint es realistisch gar nicht machbar, dass er zusätzlich für alle seine beschäftigten WBA auch noch eine fundierte und persönliche Weiterbildung in persona durchführt. Andererseits wäre aus diesem Fakt aber die Konsequenz wünschenswert, dass auch die genannten anderen ärztlichen Funktionsträger auch offiziell und formal seitens der Landesärztekammern mit der Fähigkeit zur Weiterbildung betraut werden können. Es bleibt abzuwarten, inwiefern hier die Aufsplittung der zukünftigen Facharztweiterbildung in Kompetenzbereiche, wie sie die neue MWBO vorsieht, Abhilfe schafft. Auch andere Voraussetzungen, die zur Entscheidungsgrundlage für die Vergabe von Weiterbildungsermächtigungen herangezogen werden, sollten kritisch hinterfragt werden. Bisher sind es oft noch die strukturellen Kenndaten einer Weiterbildungsstätte wie Bettenzahl, Fallzahl und das Spektrum der zur Versorgung von Patienten vorgehaltenen Fachdisziplinen innerhalb eines Krankenhauses, die auf die Entscheidung zur bzw. der Dauer einer Anerkennung als Weiterbildungsstätte Einfluss nehmen. Bei der Erteilung einer Weiterbildungsermächtigung besteht bei einem derartigen Vorgehen die Gefahr, dass möglicherweise die Qualifikation der Einzelperson nur untergeordnet hinter den rein quantitativ erfassbaren Daten zum Tragen kommt. Angaben über das Vorliegen strukturierter Weiterbildungsprogramme werden bislang von den Landesärztekammern nur vereinzelt zur Einsicht gefordert. Auch die Verlängerung bzw. Reevaluation einer einmal erteilten Ermächtigung fällt, wenn überhaupt, in der Regel erst mit der Einführung einer neuen Weiterbildungsordnung zusammen. Diese Umstände verwundern insbesondere vor dem derzeit in Deutschland grassierenden Zertifizierungs- und Akkreditierungstrend, stellt doch die Wissens- und Fertigkeitsvermittlung der WBA als ein wesentliches und daher zu objektivierendes Gut die Qualität der medizinischen Versorgung in der Zukunft sicher.

In der vorliegenden Umfrage spiegelt sich ebenfalls die als negativ empfundene Abhängigkeit der WBA von ihrem Weiterbildungsermächtigten wider. In den FTK wurden eine angebliche „Willkür“ der Weiterbildungsbevollmächtigten (so die wiederholte Originalangabe der Befragten), „fehlende objektive Strukturierungen der Weiterbildung“ sowie das vollkommene Fehlen oder aber Nichteinhalten von Weiterbildungsvereinbarungen mehrfach bemängelt. Dass diese Kritikpunkte ursächlich und inhaltlich miteinander verknüpft sind, scheint offensichtlich und unterstreicht einmal mehr den Wunsch der Weiterzubildenden nach einer unabhängigen, externen qualitativen Kontrolle ihrer Weiterbildung. Konkret hat sich mit 75,2 % die Mehrheit der WBA für eine Überprüfung der lokalen Weiterbildungsqualität durch fachlich ausgewiesene, erfahrene und v. a. auch zur Verschwiegenheit angehaltene Fachvertreter unserer wissenschaftlichen Gesellschaft (DGHNOKC) ausgesprochen. Diese könnten, nach Vorstellung der Autoren, sogar im Sinne von Ombudspersonen agieren und sich vor Ort an der jeweiligen Weiterbildungsstätte einen Überblick über die dort vorhandenen klinischen, instrumentellen und personellen Verhältnisse durch Einzelgespräche mit Vertretern aller vertretenen Hierarchieebenen verschaffen.

Das Stimmungsbild unter den Befragten der vorliegenden Arbeit ließ einen eindeutigen Trend erkennen [13]. Vordringlich wurde dabei zusammengefasst eine besser strukturierte und klarer kommunizierte Weiterbildung genannt, bei der eine unabhängige Kontrolle der Weiterbildungsqualität durch Vertreter der zuständigen Fachgesellschaft erwünscht wäre. Außerdem wurde angeregt, die Verantwortung für die Weiterbildung von der Einzelperson des WBB mehr auf das institutionalisierte und inhaltlich qualifizierte Weiterbildungsprogramm zu verlegen, ggf. mit mehreren partnerschaftlich parallel agierenden WBB. Diese Weiterbildungsprogramme sollten dabei nicht allein von den strukturellen Kenngrößen einer Abteilung abhängig sein, sondern müssten deutlich mehr als bislang auch die medizindidaktische Qualität des Weiterbildungsangebots berücksichtigen. Das Weiterbildungsprogramm selbst könnte strukturell Rotationen und auch Hospitationen zwischen den an der Ausbildung beteiligten Funktionsbereichen oder anderen medizinischen Einrichtungen vorsehen, wobei wiederum für alle Einzelbereiche klare Lern- und Ausbildungsziele definiert sein müssten [14–16]. Beispielgebend für die Umsetzung dieser Ansprüche ist dabei das existierende US-amerikanische Ausbildungssystem mit dem Accreditation Council for Graduate Medical Education (ACGME) als staatlicher Kontrolleinheit eines strukturierten gemeinsam entwickelten Weiterbildungskonzepts (Milestone Project) mit dem American Board of Otolaryngology [17].

Eine auf diese Weise angestrebte Steigerung der Weiterbildungsqualität könnte darüber hinaus idealerweise parallel mit einer expliziten Aufwertung der derzeitigen Facharztprüfungen einhergehen. Diese könnte sich nach unserer Meinung langfristig weg von der Praxis eines kollegialen, von uns als recht subjektiv eingestuften und meist unstrukturierten Gesprächs am Ende der Weiterbildungszeit hin zu einer aussagekräftigeren Prüfungsform, die eine validere, transparentere und objektivere Beurteilung des Facharztkandidaten nach seiner mindestens 5‑jährigen Weiterbildungszeit erlaubt, bewegen. Man müsste darüber diskutieren, ob durch eine Veränderung der Prüfungsmethoden auch mehr Ebenen der ärztlichen Qualifikation abgebildet und geprüft werden sollten, wie es uns im UK in allen medizinischen Fachdisziplinen vorgelebt wird. Dort beschränkt sich die Facharztprüfung nicht, wie bei uns, allein auf die Abfrage von Faktenwissen, sondern impliziert neben einem schriftlichen Prüfungsteil auch praktische Patientenuntersuchungen und die Demonstration anatomischer Präparate. Es hat sich gezeigt, dass innerhalb der in Deutschland üblichen 45-minütigen mündlichen Prüfung eine weitgehende Abbildung des HNO-Fachgebiets im Frage-Antwort-Modus nicht annähernd möglich ist. Ein solches Prüfungsergebnis kann daher, positiv wie negativ, nicht repräsentativ für den Wissens- und Fertigkeitsstand des Prüflings sein, wenn es nicht durch weitere Prüfungsleistungen ergänzt wird. Wir glauben deshalb, dass die Überprüfung auch praktischer Fertigkeiten und affektiver Lernziele im Rahmen der Facharztprüfung durchaus eine Bereicherung über den Weiterbildungsstand eines Facharztkandidaten hinaus vermitteln könnte.

Einen ersten Schritt, um konkrete Ansätze für eine Veränderung der Weiterbildung in Deutschland zu schaffen, bietet die strukturierte Erfassung von weiterbildungsrelevanten Aspekten. Dazu dienen die Ergebnisse dieser fachgruppenspezifischen Umfrage sowie die von den Ärztekammern initiierte Evaluation der Weiterbildung, welche nach 2009 erneut 2011 durchgeführt wurde. Dabei stellte sich ein positiver Trend in der Beurteilung der Weiterbildung von 2,54 auf 2,4 dar, welcher dadurch erklärt werden kann, dass die gewonnen Erkenntnisse von den teilnehmenden Weiterbildungsstätten möglicherweise schon genutzt worden sind, um sich intensiver mit dem Thema Weiterbildung auseinanderzusetzen und diese auch zu verbessern bereit war. So gaben 80 % der Weiterbildungsbefugten (*n* = 1879) an, den Befugtenbericht von 2009 klinikintern diskutiert zu haben. Dies hat die Hälfte der WBB bereits motiviert, Veränderungen an der Weiterbildung einzuleiten [19]. Die geplante kontinuierliche Fortführung von Evaluationen über die Weiterbildung sowohl fachgruppen- als auch weiterbildungsstättenspezifisch bildet deshalb nicht nur eine wesentliche objektive Grundlage, diesen Trend zu unterstützen und weitere Veränderungen in der Weiterbildung auszumachen, sondern veranlasst die WBB auf lange Sicht auch, sich dieser Thematik verbindlicher anzunehmen. Perspektivisch können durch die Integration von digitalen Applikationen zum einen Weiterbildungsinhalte jederzeit abrufbar gemacht werden, zum anderen können erreichte Weiterbildungsinhalte dokumentiert und ausgewertet werden. Damit besteht die Möglichkeit, Evaluationen zu erstellen und ein kontinuierliches Feedback an WBA und WBB zu geben. Ein erster Schritt erfolgte bereits durch die Bereitstellung einer eLogbuch-Webanwendung durch die BÄK (https://elogbuch.bundesaerztekammer.de/authentication/Account/Login). Zusätzlich wäre die Aufrechterhaltung einer turnusmäßigen jährlichen Befragung der WBA nach Schweizer Vorbild durch die BÄK ein wünschenswerter Ansatz. Dies ist bereits 2014, initiiert durch die BÄK, in einer Pilotbefragung erfolgt. Die Fortführung liegt in der Verantwortung der einzelnen Ärztekammern und soll unter Nutzung des bundesweit einheitlichen Kernfragebogens dezentral durchgeführt werden.

Betrachtet man die Veränderungen im Ausbildungssystem der medizinischen Fakultäten für Studenten seit Umsetzung der neuen Approbationsordnung im Jahr 2003 [18], so sehen wir in der praktischen Umsetzung des verbindlichen Angebots klar strukturierter Lehr- und Lernprogramme und auch der Einführung umfangreicher, repräsentativer und objektiver Prüfungen die Verhältnisse gegenüber dem gegenwärtigen Facharztweiterbildungssystem in Deutschland als klar überlegen an. Daher muss die selbstkritische Frage erlaubt sein, ob eine Zunahme von schlecht qualifizierten Bewerbern um Facharztstellen in Deutschland [19] nicht auch Ausdruck einer reformbedürftigen und, wie wir hoffen, auch verbesserungsfähigen Weiterbildungskultur ist.

### Infobox

QR-Code zum Download der vollständigen Fragebogenauswertung http://tinyurl.com/Fragenauswahl


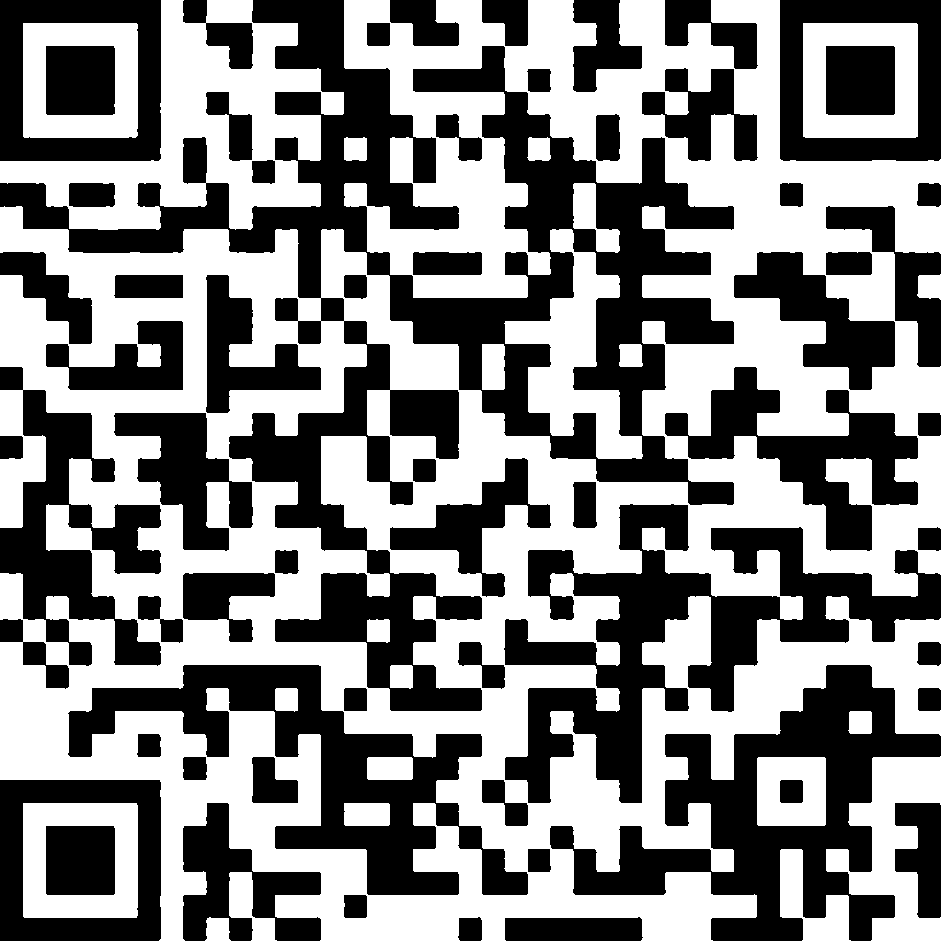


## Fazit für die Praxis

Aus den Ergebnissen der vorliegenden Arbeit lassen sich einige konkrete Maßnahmen zur Verbesserung der Weiterbildungsqualität ableiten:

- Klare Kommunikation von Lern- und Weiterbildungszielen im Rahmen der regelmäßig durchgeführten Weiterbildungsgespräche, die sowohl für den WBB als auch den WBA bindenden Charakter besitzen.
- Nutzung bereits vorhandener Instrumente für die Strukturierung der Weiterbildung wie Logbücher, Rotationspläne und ggf. externe Hospitationen.
- Ausbildung und Förderung einer auf einem konstruktiven Feedback beruhenden Kommunikationskultur, wobei sich ein solches Feedback nicht nur auf die jährlichen Weiterbildungsgespräche begrenzen, sondern sich idealerweise als kontinuierlicher Austausch zwischen dem WBB und WBA gestalten sollte.

## Caption Electronic Supplementary Material


