# Erratum zu: Die Weiterbildungssituation in der HNO-Heilkunde in Deutschland

**DOI:** 10.1007/s00106-021-01068-3

**Published:** 2021-06-10

**Authors:** J. Linke, T. Eichhorn, M. Kemper, T. Zahnert, M. Neudert

**Affiliations:** 1 Klinik für Hals‑, Nasen- und Ohrenheilkunde, Universitätsklinikum Carl Gustav Carus Dresden, Fetscher Str. 74, 01307 Dresden, Deutschland; 2 Emeritus HNO-Klinik Carl-Thiem-Klinikum, Cottbus, Deutschland

**Erratum zu:**

**HNO 2020**

10.1007/s00106-020-00838-9

Der Artikel „Die Weiterbildungssituation in der HNO-Heilkunde in Deutschland“ von J. Linke, T. Eichhorn, M. Kemper, T. Zahnert, M. Neudert wurde ursprünglich am 4. März 2020 ohne „Open Access“ online auf der Internetplattform des Verlags publiziert. Die Autoren haben sich jedoch nachträglich für eine „Open Access“-Veröffentlichung entschieden. Das Urheberrecht des Artikels wurde deshalb am 6. Mai 2021 in © Der/die Autor(en) 2020 geändert.

