# Supplementary material for: The situation of education in ENT-residency-training in Germany
Source: HNO. 2020 Mar 4;69(7):534–43. [Article in German] doi: 10.1007/s00106-020-00838-9 (PMC8233243; doi:10.1007/s00106-020-00838-9)
Supplement: Supplementary file 1 [file 106_2020_838_MOESM1_ESM.pdf]

Tabelle 1: Ergebnisdarstellung einer Fragenauswahl gegliedert nach Themenkomplexen.

[(1)] Praxis n=0; andere n=1; [(2)] 1 - stimme zu bis 6 - stimme nicht zu

Komplex	Frage	gesamt	Universitäts-kliniken	städtische Kliniken	Kliniken in privater Trägerschaft	Kliniken in kirchlicher Trägerschaft[(1)]
Weiterbildungs-stätte	Ich würde die Weiterbildungsstätte insgesamt weiterempfehlen.	n=222	n=122	n=40	n=30	n=24
	Ja	80.2%	77.9%	82.5%	83.3%	83.3%
	Nein	19.8%	22.1%	17.5%	16.7%	1.7%
	Sehen Sie an Ihrer Weiterbildungsstätte eine berufliche Perspektive?	n=221	n=124	n=40	n=30	n=26
	Ja	28.1%	25.8%	25%	36.7%	30.8%
	Ja, jedoch mit Einschränkungen.	45.2%	50.8%	40%	33.3%	42.3%
	Eher nicht.	21.7%	19.4%	27.5%	20%	26.9%
	Auf keinen Fall.	5%	4%	7.5%	10%	0%
	Die Verlängerung meines Vertrages ...	n=209	n=122	n=35	n=30	n=26
	erfolgt in regelmäßigen Abständen.	28.7%	31.1%	5.7%	36%	42.3%
	erfolgt in unregelmäßigen Abständen.	12.4%	16.4%	11.4%	4%	3.8%
	ist ungewiß.	58.9%	52.5%	82.9%	60%	53.8%
Vermittlung von Fachkompetenz	Wer betreut in erster Linie Ihre praktische Weiterbildung?	n=223	n=125	n=41	n=30	n=26
	Chefarzt	22%	8%	31.7%	66.7%	23.2%
	Oberarzt	86.5%	83.2%	90.2%	96.7%	84.6%
	Facharzt	52.5%	56.8%	36.6%	56.7%	50%
	älterer Assistenzarzt	37.7%	48%	29.3%	16.7%	26.9%
	anderer	0.9%	1.6%	0%	0%	0%
	keiner	11.7%	12.8%	17.1%	0%	11.5%
	Wie groß ist der Beitrag der Weiterbildungsstätte zur Entwicklung der folgenden Kenntnisse und Fertigkeiten?	n=222	n=124	n=41	n=30	n=26
	Erlernen von Anamneseerhebung / Patientenkommunikation	3.1±1.6	3.4±1.6	3.1±1.6	2.2±1.0	3.2±1.6
	Erlernen von Untersuchungstechniken / Befundauswertung	3.0±1.4	3.2±1.2	3.0±1.2	3.0±1.0	3.0±1.4
	Erwerb von theoretischen fachspezifischen Kenntnissen	3.2±1.3	3.2±1.3	3.6±1.3	2.8±1.4	3.2±1.3
	Erlernen von OP-Techniken	2.9±1.5	3.1±1.4	3.1±1.8	2.1±1.1	2.6±1.1
	Anleitung zum wissenschaftlichen Arbeiten	4.2±1.5	3.8±1.5	4.6±1.3	4.3±1.6	5.0±1.1
	selbstständiges Arbeiten / Übertragung von Verantwortung	2.2±1.2	2.4±1.4	2.2±1.2	2.0±0.8	1.9±1.0
Lernkultur	Mein(e) Weiterbildungsbeauftragte(r) (WBB) nimmt/nehmen sich Zeit, um mir Zusammenhänge zu erklären und meine Fragen zu beantworten.[(2)]	n=221	n=124	n=41	n=30	n=25
		3.3±1.6	3.5±1.5	3.4±1.8	2.4±1.4	2.8±1.6
	Ich erhalte regelmäßig Feedback über meine Tätigkeit.[(2)]	n=220	n=122	n=41	n=30	n=26
		4±1.5	4.3±1.4	4±1.6	3±1.5	3.7±1.3
	An meiner Weiterbildungsstätte ist es ein wichtiges Ziel eine gute Weiterbildung zu bieten.[(2)]	n=222	n=124	n=41	n=30	n=26
		3.3±1.6	3.6±1.6	3.4±1.7	2.5±1.6	3.1±1.4

**Arbeitssituation**	Ich bin insgesamt zufrieden mit der jetzigen Arbeitsbelastung.[(2)]	n=222	n=124	n=41	n=30	n=26
		3.4±1.6	3.4±1.6	3.6±1.7	2.8±1.6	3.2±1.2
	In meiner vertraglich geregelten Arbeitszeit kann ich in der Regel die Arbeit zu meiner Zufriedenheit erfüllen.[(2)]	n=222	n=124	n=41	n=30	n=26
		3.8±1.7	4.1±1.7	3.6±1.7	2.6±1.3	3.7±1.5
	In meiner vertraglich geregelten Arbeitszeit kann ich die Weiterbildung weitgehend erfüllen.[(2)]	n=222	n=124	n=41	n=30	n=26
		3.7±1.6	4±1.6	3.7±1.7	2.9±1.4	3.3±1.4
**Führungs- und Betriebskultur**	Ich bin insgesamt zufrieden mit dem jetzigen Arbeitsklima.[(2)]	n=218	n=122	n=41	n=28	n=26
		2.7±1.4	2.8±1.4	2.7±1.4	2.5±1.5	2.3±1
	Kritik an meiner Arbeitstätigkeit wird angemessen formuliert.[(2)]	n=222	n=124	n=41	n=30	n=26
		3±1.5	3.3±1.4	2.9±1.6	2.4±1.3	2.8±1.7
**Ausbildungs-struktur**	Ich bin insgesamt zufrieden mit der jetzigen Weiterbildung.[(2)]	n=222	n=124	n=41	n=30	n=26
		3±1.5	3.2±1.4	3.1±1.6	2.5±1.3	2.6±1.2
	Das durch die Weiterbildungsordnung festgelegte Gespräch mit dem Weiterbildungsbeauftragten findet statt.	n=221	n=123	n=41	n=30	n=26
	regelmäßig (jährlich)	44.8%	44.7%	34.1%	60%	42.3%
	teilweise	28.5%	22.8%	36.6%	26.7%	46.2%
	nie	26.7%	32.5%	29.3%	13.3%	11.5%
	Die in der Weiterbildungsordnung festgelegten Ziele werden in der Regelweiterbildungszeit erreicht.	n=213	n=118	n=41	n=28	n=25
	vollständig	34.3%	33.9%	24.4%	50%	32%
	teilweise	49.8%	49.2%	51.2%	42.9%	60%
	ungenügend	16%	16.9%	24.4%	7.1%	8%
	Erfolgt Ihre Ausbildung nach einem LogBuch?	n=217	n=121	n=40	n=30	n=25
	Ja,nach einem klinikinternen LogBuch.	17.1%	17.4%	12.5%	13.3%	28%
	Ja, nach einem LogBuch der europäischen Facharztweiterbildung.	25.3%	28.9%	15%	26.7%	24%
	Ja, nach einem anderen LogBuch.	6.9%	5%	0%	26.7%	0%
	Nein.	50.7%	48.8%	72.5%	33.3%	48%
	Mir wurde ein strukturierter Weiterbilungsplan zur Kenntnis gegeben.	n=217	n=124	n=39	n=29	n=24
	Ja.	21.2%	17.7%	20.5%	34.5%	20.8%
	Nein.	78.8%	82.3%	79.5%	65.5%	79.2%
	Wie oft besuchen Sie Fort- und Weiterbildungsveranstaltungen?	n=220	n=123	n=41	n=30	n=25
	wöchentlich	25.5%	35%	12.2%	16.7%	12%
	mind. 1x im Monat	23.6%	26.8%	17.1%	20%	24%
	ca. halbjährlich	37.7%	26.8%	56.1%	50%	44%
	1x im Jahr	12.7%	10.6%	14.6%	13.3%	20%
	seltener	0.5%	0.8%	0%	0%	0%
**Eigenaktivität**	Besteht die Möglichkeit zur Hospitation an anderen Weiterbildungsstätten, um hauseigen nicht vorgehaltene, in der Weiterbildungsordnung festgehaltene festgelegte Ziele (bspw. Allergologie, Phoniatrie etc.) zu erlernen?	n=217	n=122	n=39	n=29	n=26
	Ja, problemlos.	9.2%	9.8%	7.7%	10.3%	3.8%
	Ja, auf Eigeninitiative hin.	45.2%	45.9%	48.7%	48.3%	34.6%
	Nein.	45.6%	44.3%	43.6%	41.4%	61.5%
